# Spatial environmental factors predict cardiovascular and all-cause mortality: Results of the SPACE study

**DOI:** 10.1371/journal.pone.0269650

**Published:** 2022-06-24

**Authors:** Michael B. Hadley, Mahdi Nalini, Samrachana Adhikari, Jackie Szymonifka, Arash Etemadi, Farin Kamangar, Masoud Khoshnia, Tyler McChane, Akram Pourshams, Hossein Poustchi, Sadaf G. Sepanlou, Christian Abnet, Neal D. Freedman, Paolo Boffetta, Reza Malekzadeh, Rajesh Vedanthan

**Affiliations:** 1 Icahn School of Medicine at Mount Sinai, New York, New York, United States of America; 2 Digestive Disease Research Center, Digestive Disease Research Institute, Tehran University of Medical Sciences, Tehran, Iran; 3 Cardiovascular Research Center, Kermanshah University of Medical Sciences, Kermanshah, Iran; 4 New York University Grossman School of Medicine, New York, New York, United States of America; 5 Metabolic Epidemiology Branch, Division of Cancer Epidemiology and Genetics, National Cancer Institute, Bethesda, Maryland, United States of America; 6 Department of Biology, School of Computer, Mathematical, and Natural Sciences, Morgan State University, Baltimore, Maryland, United States of America; 7 Golestan University of Medical Sciences, Gorgan, Golestan, Iran; 8 Stony Brook Cancer Center, Stony Brook University, Stony Brook, New York, United States of America; 9 Department of Medical and Surgical Sciences, University of Bologna, Bologna, Italy; University of Cape Coast, GHANA

## Abstract

**Background:**

Environmental exposures account for a growing proportion of global mortality. Large cohort studies are needed to characterize the independent impact of environmental exposures on mortality in low-income settings.

**Methods:**

We collected data on individual and environmental risk factors for a multiethnic cohort of 50,045 individuals in a low-income region in Iran. Environmental risk factors included: ambient fine particular matter air pollution; household fuel use and ventilation; proximity to traffic; distance to percutaneous coronary intervention (PCI) center; socioeconomic environment; population density; local land use; and nighttime light exposure. We developed a spatial survival model to estimate the independent associations between these environmental exposures and all-cause and cardiovascular mortality.

**Findings:**

Several environmental factors demonstrated associations with mortality after adjusting for individual risk factors. Ambient fine particulate matter air pollution predicted all-cause mortality (per μg/m^3^, HR 1.20, 95% CI 1.07, 1.36) and cardiovascular mortality (HR 1.17, 95% CI 0.98, 1.39). Biomass fuel use without chimney predicted all-cause mortality (reference = gas, HR 1.23, 95% CI 0.99, 1.53) and cardiovascular mortality (HR 1.36, 95% CI 0.99, 1.87). Kerosene fuel use without chimney predicted all-cause mortality (reference = gas, HR 1.09, 95% CI 0.97, 1.23) and cardiovascular mortality (HR 1.19, 95% CI 1.01, 1.41). Distance to PCI center predicted all-cause mortality (per 10km, HR 1.01, 95% CI 1.004, 1.022) and cardiovascular mortality (HR 1.02, 95% CI 1.004, 1.031). Additionally, proximity to traffic predicted all-cause mortality (HR 1.13, 95% CI 1.01, 1.27). In a separate validation cohort, the multivariable model effectively predicted both all-cause mortality (*AUC* 0.76) and cardiovascular mortality (*AUC* 0.81). Population attributable fractions demonstrated a high mortality burden attributable to environmental exposures.

**Interpretation:**

Several environmental factors predicted cardiovascular and all-cause mortality, independent of each other and of individual risk factors. Mortality attributable to environmental factors represents a critical opportunity for targeted policies and programs.

## Introduction

Environmental factors contribute significantly to global mortality [1–4]. In 2019, environmental hazards were responsible for an estimated 11.3 million deaths, of which 5.1 million were from cardiovascular disease (CVD) [5, 6].

**Fig 1:**
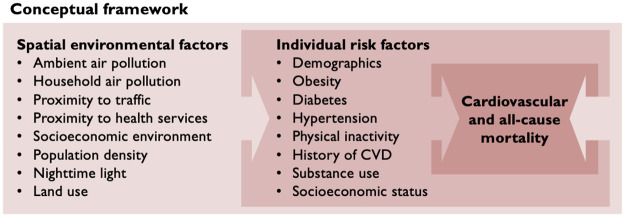
Conceptual framework. Impact of eight SEFs on individual risk factors and CV and all-cause mortality.

A growing list of environmental factors present particular risks to cardiovascular health [1–4]. Ambient fine particulate matter air pollution (PM_2.5_) from traffic, industry, fires, and dust is a risk factor for all-cause mortality, cardiovascular mortality, ischemic heart disease (IHD), and stroke [7, 8]. In 2019, ambient PM_2.5_ ranked seventh among all health risk factors for mortality, responsible for 4.14 million deaths, of which 2.47 million were from CVD [5, 6]. Similarly, household air pollution from inefficient stoves and solid fuels was responsible for 2.31 million deaths (1.07 million from CVD) [5, 6]. Proximity to traffic pollution and noise is associated with increased rates of adverse CVD events, particularly IHD and stroke [4, 7, 9]. Distance to health care services affects access to preventive care, tertiary care, and emergent percutaneous coronary interventions (PCI) [10, 11]. Socioeconomic environment is an independent risk factor for CVD, even after controlling for individual socioeconomic status [12]. Population density has been shown to be both positively and negatively correlated with CVD in different environments [13, 14]. Exposures to artificial light at night cause circadian dysregulation and have been associated with ischemic heart disease outcomes [4, 15, 16]. Finally, land use (e.g., greenspaces, mixed land use) may predict CVD through association with physical activity, social engagement, and access to health services [17, 18]. Together, these variables act directly and indirectly to precipitate cardiovascular disease and mortality (Fig 1).

Understanding the relationships between environmental risk factors and health is a critical step towards designing targeted policies and programs to reduce the immense burden of attributable disease. To date, most investigations of environmental factors have studied *single* risk factors in *high-income* settings. We therefore developed a spatial environmental model to provide a better understanding of the independent associations between multiple spatial environmental factors and mortality, within a low-income population in the middle-income country of Iran.

## Methods

### Study setting

We analyzed data of participants enrolled in the Golestan Cohort Study (GCS) in Golestan Province, Iran, a middle-income country with diverse ethnicities and lifestyles. In both Iran and Golestan, CVD is the leading cause of death and disability [19].

### Study participants

The GCS enrolled 50,045 individuals (28,811 females and 21,234 males) across northeastern Golestan from 2004 to 2008 [20]. Participants ranged in age from 40 to 75 years in order to capture individuals with higher rates of non-communicable disease, particularly esophageal cancer. Approximately 80% were enrolled from 326 rural villages ranging in size from 20 to 150 residents. The remaining 20% were selected randomly from Gonbad City, the second-largest city in Golestan with a population of approximately 130,000. Exclusion criteria were: unwillingness to participate for any reason; being a temporary resident; or having a previous diagnosis of upper gastrointestinal cancer. Among those selected to enroll, participation rates were approximately 80% for women and 65% for men. Participants were followed-up actively every 12 months with a follow-up success rate was 99%. Study methods were approved by ethics review committees of the Tehran University of Medical Sciences, the International Agency for Research on Cancer, and the National Cancer Institute. All participants signed a written informed consent at enrollment. The full study protocol is publicly available [20].

### Individual characteristics

Individual baseline characteristics were collected at time of enrollment in the GCS [20]. Participants were interviewed by a physician in their native language and completed a detailed lifestyle questionnaire and physical exam. The following covariates were included in our analysis: age, sex, ethnicity, marital status, education, socioeconomic status, waist and hip circumference, physical activity, medical history (IHD, stroke, diabetes, hypertension), and substance use (tobacco, alcohol, and opium). Details on the socioeconomic status score can be found in S1 File.

At baseline, individuals enrolled in the GCS were, on average, 52.1 years of age (SD 8.9 years) and 58% female. The majority was married (88%) and illiterate (70%). A minority had a history of IHD or stroke (6%), diabetes (7%), hypertension (20%), tobacco use (22%), opium use (17%), or alcohol use (3%). Three-quarters were of Turkmen ethnicity and the remainder were of other ethinc groups (e.g., Persians, Baluchis, Qizilbash), consistent with the prevalence of ethnic groups in the sampled region. The derivation and validation cohorts were similar in the distribution of risk factors (Table 1).

**Table 1:** Individual characteristics.

Characteristic	Level	Derivation cohort (n = 45,052)	Validation cohort (n = 5003)
**Average age at baseline**	Years, mean ± SD	52.1 ± 8.9	52.1 ± 9.0
**Sex**	Female	25864 (57%)	2947 (59%)
	Male	19178 (43%)	2056 (41%)
**Ethnicity**	Turkmen	33536 (75%)	3717 (74%)
	All other ethnicities	11420 (25%)	1273 (26%)
**Residence**	Urban	9039 (20%)	993 (20%)
	Rural	36003 (80%)	4010 (80%)
**Marital status**	Married	39514 (88%)	4376 (88%)
	All others	5430 (12%)	610 (12%)
**Education level**	No education/Illiterate	31600 (70%)	3518 (70%)
	All others	13442 (30%)	1485 (30%)
**Socioeconomic status**	MCA quartile 1 (lowest)	12491 (28%)	1447 (29%)
	MCA quartile 2	10066 (22%)	1078 (22%)
	MCA quartile 3	11340 (25%)	1246 (25%)
	MCA quartile 4 (highest)	11145 (25%)	1232 (25%)
**Waist circumference**	Centimeters, mean ± SD	95.3 ± 13.7	95.2 ± 13.7
**Hip circumference**	Centimeters, mean ± SD	99.5 ± 9.4	99.4 ± 9.3
**Physical activity**	Tertile 1 (least active)	14912 (33%)	1670 (33%)
	Tertile 2	13894 (31%)	1545 (31%)
	Tertile 3 (most active)	14354 (32%)	1574 (31%)
**History of IHD or CVA**	Yes	2737 (6%)	314 (6%)
	No	42305 (94%)	4689 (94%)
**History of diabetes**	Yes	3110 (7%)	344 (7%)
	No	41932 (93%)	4659 (93%)
**History of hypertension**	Yes	8866 (20%)	1009 (20%)
	No	36176 (80%)	3994 (80%)
**Tobacco**	Current	7844 (17%)	862 (17%)
	Former	1927 (4%)	221 (4%)
	Never	35271 (78%)	3920 (78%)
**Opium use**	Ever	7622 (17%)	865 (17%)
	Never	37420 (83%)	4138 (83%)
**Alcohol use**	Ever	1520 (3%)	189 (4%)
	Never	43522 (97%)	4814 (96%)

Individual characteristics of derivation and validation cohorts. Characteristics were recorded at time of enrollment. Medical comorbidities were self-reported. There was good similarity in the distribution of individual characteristics between the derivation and validation cohorts. MCA = multiple component analysis index of individual socioeconomic status. IHD = ischemic heart disease. CVA = cerebrovascular accident (stroke).

### Mortality data

In the GCS, nearly 100% of deaths are captured via documents collected from participants, hospital files, and verbal autopsy questionnaires, which are reviewed by at least two independent internists to define diagnoses according to the *International Classification of Diseases*, *10*^*th*^ *Revision* (ICD-10) codes [21, 22]. All-cause mortality included all reported deaths. Cardiovascular mortality included deaths attributable to IHD (ICD-10 codes I20-25), cerebrovascular disease (I60-69), cardiac arrest (I46), congestive heart failure (I50), hypertensive diseases (I10-15), chronic rheumatic heart diseases (I05-09), pulmonary heart disease (I26-28), and other cardiovascular system diseases not otherwise specified. Complete case analysis was used for missing data.

### Individual geocodes

Each individual was assigned a geocode (latitude and longitude) based on residence location. Details on geocode assignment can be found in S1 File.

### Spatial environmental factors

We developed exposure variables for eight prespecified spatial environmental factors (SEFs) using a combination of GCS data and publicly available datasets [20, 23–28]. These variables were chosen based on a review of the literature on environmental risks for cardiovascular disease [1–4], as well as data availability. The SEFs were: ambient air pollution [24, 25], household fuel use and ventilation [20], socioeconomic environment [23], proximity to traffic (within 100m of a minor highway or within 500m of a major highway) [20], distance to percutaneous coronary intervention centers [20], population density [26], nighttime light exposure [27], and land use [28]. Environmental exposures were assigned according to year of enrollment. Some potential environmental hazards were not included because no data was available for the study region (e.g., noise pollution; toxins in water and food). All SEFs were included in the final multivariable model. Data sources and related methodologies are summarized in **S1 Table in** S1 File. Please see Data Sharing Statement for information on how to access GCS-specific data.

### Derivation and validation cohorts

The GCS dataset was randomized into derivation and validation cohorts, stratified to ensure mortality was balanced in both groups, following established methodology [29]. The derivation cohort (90%) was used to construct the multivariable spatial environmental model; the validation cohort (10%) was used subsequently to test the model’s predictive value. The derivation cohort contained 45,042 individuals and 5996 deaths, of which 2733 were cardiovascular deaths. The validation cohort contained 5003 individuals and 655 deaths, of which 286 were cardiovascular deaths. A summary of the study design is shown in **S1 Fig in** S1 File.

### Statistical analyses

We developed spatial environmental survival models to study the association of each SEF on the hazard of mortality, for both all-cause and cardiovascular mortality. All eight SEFs were tested simultaneously in the multivariable survival model, in order to adjust for each other. Additionally, we adjusted for common individual risk factors, including sex, age, individual socioeconomic status, anthropometric measures, and history of cardiovascular disease, hypertension, diabetes, and smoking. We used a spatial random effects survival model [30–32] (e.g., shared frailty model) to model time to mortality. For an individual *j* in geocode *i* with censoring time *t* given covariates *x*_*ij*,_, the survival function in the frailty model is:

$${\text{S}}_{{\text{x}}_{\text{ij}}}\left(\text{t}\right)={\text{S}}_{0}\left(\text{t}\right){\text{exp}}^{{\text{x}}_{\text{ij}}}{}^{\;\beta +\nu_{\text{i}}}.$$

Here, *β* is a vector of regression coefficients and *νi* is a frailty parameter (or the random effect) for geocode *i* and is assumed to have a gaussian distribution. Environmental exposures demonstrated low correlation at the geocode level reducing the risk of collinearity and model overfitting. Computations were performed in R (packages ‘survival’ and ‘spBayesSurv’) [33]. We report the exponentiated coefficients as the estimate of hazard ratio along with 95% confidence interval of the estimates.

To account for spatial dependence, as a sensitivity analysis we also fitted the Bayesian survival models that adjust for spatial autocorrelation based on distance between geocodes [34, 35]. Gaussian random field priors were specified on the frailty parameters to allow for autocorrelation between neighboring geocodes.

We validated the model using recent methodologies for external validation [36]. First, we used a chi-squared test to determine whether the addition of SEFs improved the model’s goodness-of-fit beyond traditional risk factors in the derivation cohort. Next, time-dependent area-under-the-curve analysis was used to test how well the spatial frailty model predicted all-cause and cardiovascular mortality in the novel validation cohort.

Finally, we calculated the population attributation fraction (PAF) for all categorical predictors in the multivariable model. PAFs incorporate both the hazard and prevalence of a risk factor to assess the fraction of total disease risk in the population that would be eliminated (or added) if the risk factor were eliminated from the population (i.e., if all individuals with that risk factor were moved to the reference category) [37].

### Patient and public involvement

Local residents, physicians, elders, religious leaders, and university physicians were deeply involved in the design and implementation of the Golestan Cohort Study. Details can be found in S1 File.

## Results

### Distribution of spatial environmental factors

**Fig 2:**
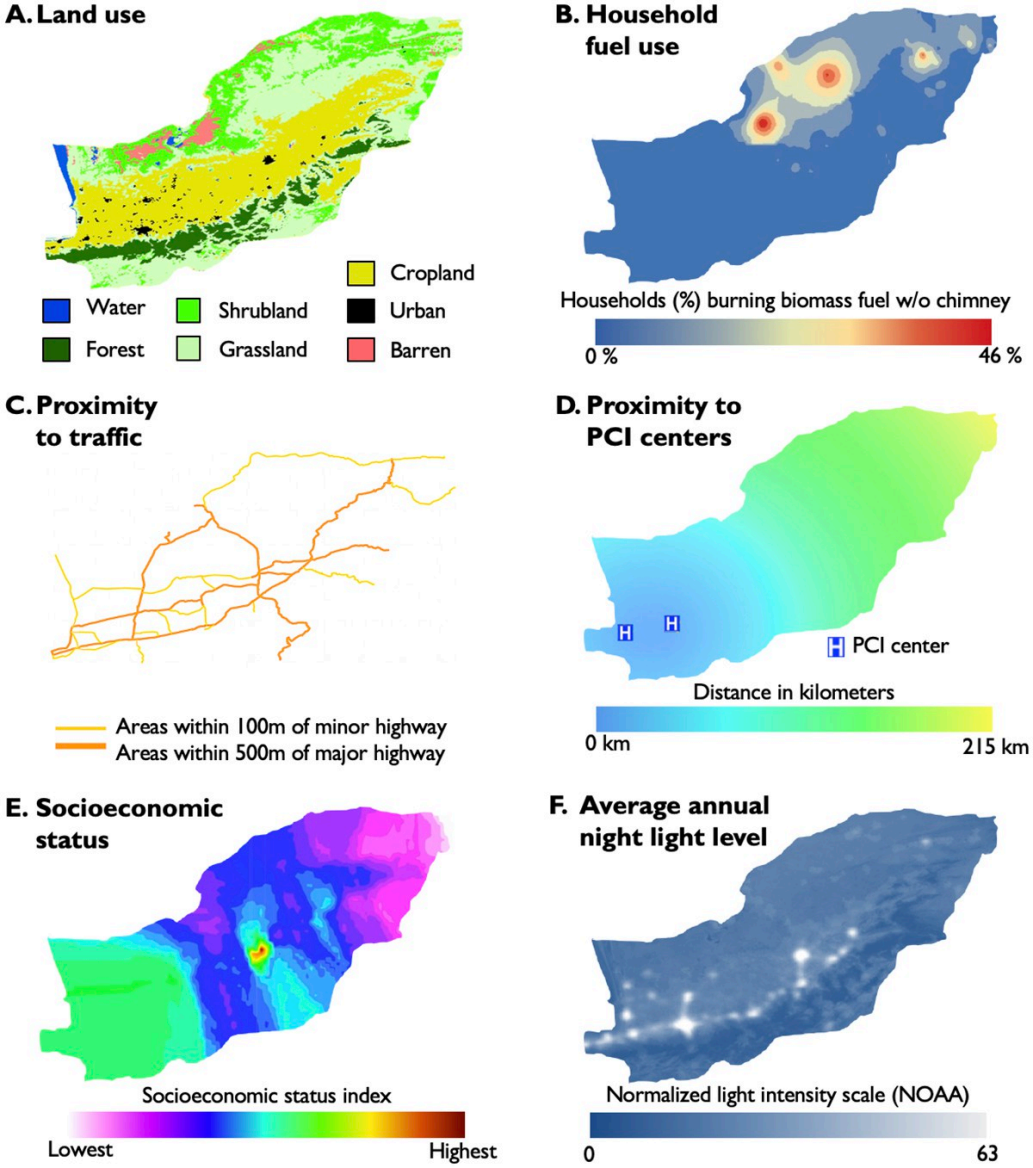
Spatial models for six spatial environmental factors (SEFs) across Golestan Province, Iran. These models were used to assign environmental exposures to individuals based on their location of residence. Methodologies are described in **S1 Table in** S1 File. **A.** Land use based on satellite imagery [28]. **B.** Proportion of households burning biomass fuels without a chimney. Estimates derived from Gaussian process regression (kriging) of average fuel use patterns for each village geocode [20]. **C.** Proximity to traffic, defined as within 100m of a minor highway or within 500m of a major highway [20]. **D.** Proximity to percutaneous coronary intervention centers [20]. **E.** Average socioeconomic status index score. Estimates derived from kriging of median socioeconomic score for each geocode [23]. **F.** Average annual intensity of light-at-night using satellite imagery [27]. Models developed in ArcGIS [38]. All displayed data is either in the public domain, collected by the investigators, or for illustrative purposes only.

Across all geocodes, average annual fine particulate matter air pollution exposures were 33.5 μg/m^3^ for the 5 years prior to enrollment (167.7 μg/m^3^ cumulatively, SD 17.5 μg/m^3^). Most households burned kerosene fuel (71%), of which 42% had a chimney for ventilation. A total of 7% of households used biomass fuels (typically wood or dung burned indoors for cooking or heating), of which 81% had a chimney. The remainder of households providing responses used either gas (12%) or mixed fuels (9%). One third of participants (34%) lived close to major highways. Distances to the nearest percutaneous coronary intervention center averaged 92.2 km (SD 37.4 km). The socioeconomic status score varied with location, with lower scores concentrated in the northeast. Local population density averaged 1732 persons per square kilometer (SD 3069). The intensity of nighttime light averaged 22.7 (SD 22.2) on a NOAA intensity metric. Both population density and nighttime light exposure were highest in Golestan’s central agricultural valley. Most households were located amidst cropland (57%) or urban settings (25%), with the remainder located among shrubland (9%), grassland (9%), or barren earth (<1%). The derivation and validation cohorts were similar in the distribution of all SEFs (Table 2). The spatial distribution of the SEFs is illustrated in Fig 2.

**Table 2:** Individual exposures to spatial environmental factors.

Characteristic	Level	Derivation cohort (n = 45,052)	Validation cohort (n = 5003)
**Average annual PM**_**2.5**_ **cumulative for 5 years prior to enrollment**	μg/m^3^ (mean ± SD)	167.7 ± 17.5	167.7 ± 17.5
**Household fuel use**	Biomass without chimney	597 (1%)	60 (1%)
	Biomass with chimney	2575 (6%)	306 (6%)
	Kerosene without chimney	18666 (41%)	2083 (42%)
	Kerosene with chimney	13252 (29%)	1470 (29%)
	Gas	5442 (12%)	592 (12%)
	Mixed fuels	4018 (9%)	429 (9%)
**Proximity to traffic**	Within 100m of minor highway	10324 (23%)	1113 (22%)
	Within 500m of major highway	14729 (32%)	1599 (31%)
**Proximity to PCI center**	Km (mean ± SD)	92.2 ± 37.4	92.0 ± 37.3
**Average neighborhood socioeconomic status**	MCA score (mean ± SD)	0.005 ± 0.12	0.006 ± 0.12
**Population density**	Persons/km^2^ (mean ± SD)	1730.9 ± 3068.9	1742.5 ± 3079.1
**Average annual light-at-night intensity value**	NOAA metric (mean ± SD)	22.7 ± 22.2	22.9 ± 22.2
**Land use**	Cropland	25581 (57%)	2862 (57%)
	Urban	11089 (25%)	1274 (25%)
	Shrubland	4196 (9%)	436 (9%)
	Grassland/savanna	4058 (9%)	418 (8%)
	Barren	118 (0%)	13 (0%)

Individual exposures to environmental risk factors in the derivation and validation cohorts. Exposures were assigned according to spatial models developed for each environmental risk factor (Fig 2). Three different measures of ambient fine particulate matter air pollution (PM_2.5_) were tested. There was good similarity in the distribution of spatial environmental risk factors between the derivation and validation cohorts. PCI = percutaneous coronary intervention center. MCA = multiple component analysis index of individual socioeconomic status (range: -0.46 to +0.49). NOAA = National Oceanic and Atmospheric Administration.

### Mortality data

In the derivation cohort (n = 45,042), there were 2733 cardiovascular deaths and 5996 all-cause deaths. In the validation cohort (n = 5003), there were 286 cardiovascular deaths and 655 all-cause deaths. The mean follow-up time in the derivation and validation cohorts was 10.2 years and 10.9 years, respectively. Mean time to all-cause death in the derivation and validation cohorts was 6.2 years and 6.1 years, respectively. Mean time to cardiovascular death in both cohorts was 5.8 years. In both derivation and validation cohorts, all-cause mortality was 13% and cardiovascular mortality was 6% over the follow-up period.

### Individual characteristics predict mortality

Individual characteristics predicted all-cause and cardiovascular mortality in the derivation cohort (Table 3). The following variables demonstrated *increased* hazard for both all-cause and cardiovascular mortality: older age, male gender, being unmarried, lower socioeconomic status, illiteracy, higher waist circumference (adjusted for hip circumference), lower hip circumference (adjusted for waist circumference), lower physical inactivity, tobacco use, opium use, and history of hypertension, diabetes, IHD, and stroke. Turkmen ethnicity was associated with increased hazard for all-cause mortality, but not for cardiovascular mortality.

**Table 3:** Hazard ratios for individual risk factors.

Risk factor	Hazard ratio [95% CI]	
	Cardiovascular mortality	All-cause mortality
Age, per 10 years	1.98 [1.89, 2.08]	2.02 [1.95, 2.08]
Male sex (reference = female)	1.76 [1.59, 1.97]	1.69 [1.57, 1.82]
Turkmen ethnicity (reference = all others)	1.06 [0.96, 1.16]	1.12 [1.05, 1.19]
Married (reference = all others)	0.87 [0.79, 0.97]	0.85 [0.79, 0.91]
Illiteracy (reference = all others)	1.19 [1.06, 1.34]	1.13 [1.05, 1.22]
SES (reference = quartile 1)		
Quartile 2	0.92 [0.83, 1.02]	0.85 [0.79, 0.92]
Quartile 3	0.76 [0.68, 0.85]	0.79 [0.73, 0.85]
Quartile 4	0.70 [0.62, 0.80]	0.70 [0.64, 0.77]
Physical activity (reference = tertile 3)		
Tertile 1	1.41 [1.26, 1.57]	1.36 [1.26, 1.45]
Tertile 2	1.12 [0.99, 1.27]	1.08 [0.99, 1.17]
Waist circumference (per 1 cm increase)	1.02 [1.01, 1.02]	1.01 [1.01, 1.10]
Hip circumference (per 1 cm increase)	0.99 [0.97, 0.98]	0.98 [0.97, 0.98]
History of IHD or CVA (reference = none)	2.10 [1.89, 2.33]	1.55 [1.43, 1.68]
History of diabetes (reference = none)	1.92 [1.72, 2.14]	1.90 [1.76, 2.06]
History of hypertension (reference = none)	1.93 [1.77, 2.10]	1.47 [1.38, 1.56]
Current tobacco use (reference = never)	1.29 [1.15, 1.44]	1.32 [1.22, 1.42]
Former tobacco use (reference = never)	1.23 [1.05, 1.43]	1.18 [1.06, 1.32]
History of opium use (reference = never)	1.45 [1.32, 1.61]	1.51 [1.41, 1.62]
History of alcohol use (reference = never)	1.01 [0.84, 1.22]	1.08 [0.96, 1.22]

Hazard ratios for cardiovascular and all-cause mortality associated with individual risk factors in the multivariable spatial environmental model. SES = socioeconomic status. IHD = ischemic heart disease. CVA = cerebrovascular accident (stroke).

### Spatial environmental factors predict mortality

We identified several SEFs that predicted mortality in the multivariable derivation model (Table 4). (Univariate estimates are provided in **S2 Table in** S1 File).

**Table 4:** Hazard ratios for eight spatial environmental factors.

Spatial environmental factor	Hazard ratio [95% CI]	
	Cardiovascular mortality	All-cause mortality
**Ambient PM**_**2.5**_ (μg/m^3^, reference = quartile 1)		
Quartile 2	1.11 [0.99, 1.25]	1.08 [0.99, 1.16]
Quartile 3	1.09 [0.98, 1.24]	1.09 [1.01, 1.18]
Quartile 4	1.17 [0.98, 1.39]	1.20 [1.07, 1.36]
**Household fuel use** (reference = gas)		
Biomass without chimney	1.36 [0.99, 1.87]	1.23 [0.99, 1.53]
Biomass with chimney	1.11 [0.88, 1.39]	1.05 [0.89, 1.22]
Kerosene without chimney	1.19 [1.01, 1.41]	1.09 [0.97, 1.23]
Kerosene with chimney	0.98 [0.83, 1.17]	0.98 [0.87, 1.11]
Mixed fuel use	0.98 [0.87, 1.09]	1.02 [0.87, 1.20]
**Proximity to traffic** (reference = no)	1.13 [1.01, 1.27]	1.04 [0.96, 1.12]
**Distance to PCI center** (per 10 km)	1.02 [1.004, 1.03]	1.01 [1.004, 1.02]
**Neighborhood SES score**	0.75 [0.45, 1.24]	0.79 [0.56, 1.14]
**Population density** (per 1000 persons/km^2^)	0.98 [0.96, 1.01]	0.99 [0.98, 1.02]
**Light at night intensity**	1.00 [0.99, 1.01]	1.00 [0.99, 1.01]
**Land use** (reference = urban)		
Cropland	0.93 [0.76, 1.12]	1.07 [0.94, 1.22]
Shrubland	1.03 [0.82, 1.30]	1.05 [0.89, 1.24]
Grassland/savanna/barren	0.91 [0.72, 1.15]	1.02 [0.87, 1.21]

Hazard ratios for cardiovascular and all-cause mortality associated with eight SEFs in the derivation model, after adjustment for individual risk factors. SES = socioeconomic status.

In the multivariable model, exposure to outdoor air pollution predicted all-cause mortality (per μg/m^3^, HR 1.20, 95% CI 1.07 to 1.36) and cardiovascular mortality (per μg/m^3^, HR 1.17, 95% CI 0.98 to 1.39).

Exposure to household air pollution also demonstrated predictive power. Specifically, the use of household kerosene fuel use without a chimney compared to gas predicted cardiovascular mortality (HR 1.19, 95% CI 1.01 to 1.41) and all-cause mortality (HR 1.09, 95% CI 0.97 to 1.23). Additionally, the use of biomass fuel without a chimney compared to gas predicted both cardiovascular mortality (HR 1.36, 95% CI 0.99 to 1.87) and all-cause mortality (HR 1.23, 95% CI 0.99 to 1.53).

Greater distance to PCI centers also was associated with increased hazard of both cardiovascular mortality (per 10 km, HR 1.02, 95% CI 1.004 to 1.03) and all-cause mortality (per 10 km, HR 1.01, 95% CI 1.004 to 1.02). Proximity to traffic also increased the hazard of cardiovascular mortality (HR 1.13, 95% CI 1.01 to 1.27).

The remaining SEFs—neighborhood socioeconomic status, local population density, nighttime light, and land use—did not demonstrate relationships with either all-cause or cardiovascular mortality.

### Spatial environmental factors add predictive power

The addition of the eight SEFs improved the predictive power of the model beyond traditional risk factors. Goodness of fit statistics (chi-squared statistics and corresponding p-values) were used to compare the derivation model incorporating environmental variables to the reduced model. For both cardiovascular and all-cause mortality, the addition of SEFs improved goodness of fit (*chi-squared(df)*: 57.5(16), p<0.001 for cardiovascular mortality; 54.3(16), p<0.001 for all-cause mortality).

### Stratification by sex

A sex-stratified analysis found no effect modification of the relationship between SEFs and either all-cause or cardiovascular mortality.

### Adjusting for spatial autocorrelation

After adjustments for spatial autocorrelation, there was no meaningful change in the magnitude or direction of the point estimates for the eight SEFs. The spatial autocorrelation model was consistent with the original frailty estimates.

### Model validation

The model incorporating SEFs was tested on a novel validation cohort. In this validation cohort, the spatial frailty model effectively predicted mortality with time-dependent areas-under-the-curve of 0.76 for all-cause mortality and 0.81 for cardiovascular mortality.

### Population attributable fractions

PAFs for common individual risk factors were characteristically large, including hypertension (CV mortality 0.42; all-cause mortality 0.25), diabetes (CV 0.41; all-cause 0.35), and current tobacco use (CV 0.16; all-cause 0.12). PAFs were substantial for SEFs, including ambient PM_2.5_ (highest quartile, CV mortality 0.14; all-cause mortality 0.15), biomass fuel use without chimney (CV 0.24; all-cause 0.15), kerosene fuel use without chimney (CV 0.15; all-cause 0.07), and proximity to traffic (CV 0.11; all-cause 0.03). These PAFs are illustrated in **S2 Fig in** S1 File, along with PAFs for several common individual risk factors. PAFs for all categorical predictors can be found in **S3 Table in** S1 File.

## Discussion

### Statement of principal findings

In this analysis of the Golestan Cohort Study, several SEFs predicted cardiovascular or all-cause mortality in the spatial survival model after adjusting for individual risk factors. When applied to a novel validation cohort, the model effectively predicted both cardiovascular and all-cause mortality.

#### Ambient air pollution

In our model, ambient air pollution levels were associated with all-cause and cardiovascular mortality after adjusting for other environmental risk factors in the multivariable model. Additionally, PAFs identified a large burden of mortality attributable to ambient air pollution. This is consistent with the existing literature demonstrating associations between ambient PM_2.5_ and both cardiovascular and all-cause mortality [3–7].

#### Household air pollution

We found that household kerosene fuel use without a chimney was associated with both cardiovascular mortality and all-cause mortality. This is consistent with a previous study of the GCS that found associations between cumulative kerosene exposure and both cardiovascular and all-cause mortality [39]. Additionally, we observed modest evidence of an association between the use of biomass fuel without a chimney and both cardiovascular and all-cause mortality. This is consistent with the existing literature on the cardiovascular effects of indoor burning of solid biomass [3, 5, 6]. Additionally, PAF calculations illustrate a high burden of cardiovascular and all-cause mortality attributable to biomass and kerosene fuel burning.

#### Proximity to traffic

In our model, residence close to highways increased the hazard of cardiovascular mortality. Although this hazard ratio was only 1.13, many individuals live near major roadways, resulting in a PAF of 0.11 for cardiovascular mortality. Importantly, this association was observed after adjusting for ambient air pollution and socioeconomic environment in the multivariable model. This suggests that the calculated hazard may be due chiefly to traffic-related noise or to another unmeasured variable associated with proximity to traffic. These findings are consistent with prior studies that have demonstrated associations between proximity to traffic and cardiovascular events, particulary IHD and stroke [7, 9].

#### Distance to health care services

We found that greater distance to PCI centers increased the hazard of both cardiovascular and all-cause mortality. These results align with a previous cohort study demonstrating that distance to hospital is an independent predictor of mortality among patients with incident MI in the community [11].

Several other SEFs did not demonstrate clear relationships in the multivariable model: neighborhood socioeconomic status, local population density, nighttime light, and land use. Low socioeconomic status neighborhood environment previously was shown to predict cardiovascular events [12, 40], and did so in our univariate model for cardiovascular mortality, but not in the multivariable environmental model. This suggests that socioeconomic environment may be a proxy for other environmental risk factors (e.g., air pollution) that were included in our model.

### Strengths and limitations

A key strength of our model was the simultaneous testing of multiple environmental risk factors. To date, most studies of environmental risk look at a *single* environmental risk factor against a background of individual characteristics. This can lead to confounding by other unmeasured environmental factors. In this study, we incorporate a diversity of spatially resolved SEFs in a prospective model predicting cardiovascular mortality. Additionally, the study was performed in a rural, low-income setting, helping to bridge a gap in the medical literature.

This study also benefitted from the use of the GCS dataset, which included a large sample size and excellent follow-up rate, as well as systematic methods to identify mortality and attributable causes of death. Additionally, our spatial random effects survival model controlled for possible spatial dependency in the data.

Our study had several limitations. First, exposures were assigned according to village- or neighborhood-level geocodes, rather than specific home addresses (due to human subjects-related privacy considerations). This could result in exposure misclassification and bias our findings towards the null. However, given the small sizes of villages, we estimate the average distance between assigned geocodes and true home address to be less than 500 meters. With the exception of proximity to traffic, none of our modeled environmental exposures vary dramatically over this distance.

Second, the spatial environmental factors in our model were assessed at the year of enrollment. This may misclassify exposure for participants exposed to SEFs prior to enrollment, for participants migrating to new locations with different exposures, or if the exposure varied over time. Similarly, given that environmental exposures were assigned at enrollment, our model does not account for acute exposures that may result in acute cardiovascular events (e.g. acute air pollution exposure triggering coronary plaque rupture). Again, we would expect these factors to bias our findings towards the null.

Third, household fuel use and ventilation were used as proxies for air pollution exposure. Although not optimal, this method has been used previously to study air pollution exposures in Golestan [39], as well as in studies on the association between household air pollution and childhood mortality [41].

Fourth, the socioeconomic environment variable used wealth indices from study participants and could be inaccurate if the mean wealth index at each geocode is not representative of the community.

Finally, although we have adjusted for eight SEFs in the environmental model, there remains the possibility of residual confounding from unmeasured SEFs (e.g. climate; temperature variation; noise pollution; toxins in water and food) and individual risk factors (e.g. dyslipidemia).

## Conclusions

We tested the prospective associations between eight environmental factors and cardiovascular and all-cause mortality in a large cohort in a low-income setting. Our findings demonstrate that the burden of disease attributable to the environment may be as large as traditional cardiovascular risk factors, and thus represents a critical opportunity for targeted policies and programs. Furthermore, these findings illustrate the utility and feasibility of incorporating environmental data in survival models, even in low-income settings.

A growing literature illustrates how health care providers, governments, and charities can identify and intervene on environmental exposures at the individual and population levels [8, 42, 43]. We anticipate that these findings and analytic approach will stimulate further studies to promote better health for populations and the environment worldwide.

## Supporting information

S1 File (DOCX)

## Abbreviations

CVD: Cardiovascular disease
GCS: Golestan Cohort Study
IHD: Ischemic heart disease
MCA: Multiple correspondence analysis
PCI: Percutaneous coronary intervention
SEF: Spatial environmental factor
SPACE: Spatial assessment of cardiovascular events
