# Supplementary material for: Spatial environmental factors predict cardiovascular and all-cause mortality: Results of the SPACE study
Source: PLoS One. 2022 Jun 24;17(6):e0269650. doi: 10.1371/journal.pone.0269650 (PMC9231727; doi:10.1371/journal.pone.0269650)
Supplement: S1 File — (DOCX) [file pone.0269650.s001.docx]

**S1 File**

**Methods**

**Socioeconomic status score**

Socioeconomic status was defined as a composite wealth score derived by applying multiple correspondence analysis (MCA) to data on household assets (e.g. personal car, washing machine) [1].

**Spatial environmental factors**

We developed exposure variables for eight prespecified spatial environmental factors (SEFs) using a combination of GCS data and publicly available datasets. Environmental exposures were assigned according to year of enrollment. Data sources and related methodologies are summarized below and in **S1** **Table**.

*Ambient air pollution*: Ambient air pollution exposures were assigned to individuals based on average annual estimates of ground-level PM_2.5_ in μg/m^3^ at their assigned geocode. Estimates were derived by combining aerosol optical depth retrievals from the NASA MODIS, MISR, and SeaWIFS instruments with the GEOS-Chem chemical transport model, and subsequently calibrated to global ground-based observations of PM_2.5_ using geographically weighted regression [2, 3]. Estimates are available at a resolution of 0.01° × 0.01° of latitude and longitude (approximately one kilometer square). Exposures were quantified as cumulative air pollution exposure for the 5 years prior to enrollment, and divided into quartiles for analysis.

*Household fuel use and ventilation*: Household fuel use was documented on enrollment in the GCS. Fuel use was categorized as biomass fuel, kerosene fuel, gas, or mixed fuel use. The presence or absence of a chimney for ventilation was also documented.

*Socioeconomic environment*: To estimate socioeconomic environment at each geocode, we used the median MCA score for all individuals in the village or urban neighborhood assigned to that geocode [1]. Individual MCA score was also used as a covariate in the analysis.

*Proximity to traffic*: We developed a map of all paved high-speed highways in Golestan. Highways were divided into major highways (multi-lane connecting major population centers) and minor highways (two-lanes connecting smaller population centers). The radius of exposure is larger for major roads with higher traffic throughput [4]. Accordingly, individuals were designated as living in proximity to traffic if their assigned geocode fell within 100 meters of a minor highway or within 500 meters of a major highway. Individuals not meeting either criterion were designated as not living within proximity to traffic.

*Distance to health care services*: Each individual was assigned a distance to the nearest center with capabilities for PCI, calculated as the straight-line distance from the health facility to an individual’s geocode [5].

*Population density*: Local population density (persons per square kilometer) was assigned to each individual according to estimates of population density on year of enrollment at the individual’s geocode. Estimates were provided by the LandScan platform, which uses spatial data, satellite imagery, and multi-variable dasymetric modeling to estimate population density at a resolution of one square kilometer [6].

*Nighttime light exposure*: Average annual nighttime light exposure was assigned to each individual according to estimates at the individual’s geocode. Estimates were provided by the National Oceanic and Atmospheric Administration Centers for Environmental Information, which uses composite satellite imagery from the Defense Meteorological Satellite Program – Operational Linescan System to provide light intensity values at a resolution of 30 arc seconds square [7].

*Land use*: Land use at place of residence was assigned to each individual according to land use data on year of enrollment at the individual’s geocode. Estimates were provided by the USGS global land cover survey, which employs satellite imagery to define local ecology at a resolution of 500 meters square [8]. Individuals were assigned to one type of land use, as per the USGS Land Cover NLCD 92 definitions [8]: urban, cropland, open shrubland, grassland/savanna, and barren.

**Individual geocodes**

Each individual was assigned a geocode (latitude and longitude) based on residence location. Geocodes were assigned to individuals living in rural villages according to the geographic center of their village. Since most rural villages have fewer than 100 residents, most individual residences were close to this geographic center. For individuals living in Gonbad City, geocodes were assigned according to the location of their primary health center, typically within a few blocks of their residence. In the Golestan Cohort, each of 40,013 individuals living in rural villages was assigned one of 337 village geocodes, while each of 10,032 individuals living in Gonbad City was assigned one of 286 urban geocodes.

**Patient and public involvement**

Prior to developing the study, the research group informed local authorities, university officials, physicians, elders, and religious leaders about the aims of the Golestan Cohort Study. Local residents and physicians were consulted about various exposures, as well as appropriate ways of asking questions. All interviews, data, and sample collection were performed by trained local residents. Results of blood testing were reported to participants. All participants received cohort membership cards, which allowed them to use certain medical services for free in a clinic established by the research team. Salient results are routinely shared with the Ministry of Health, university officials, local physicians, and the public.

**Supporting information: figures & tables**


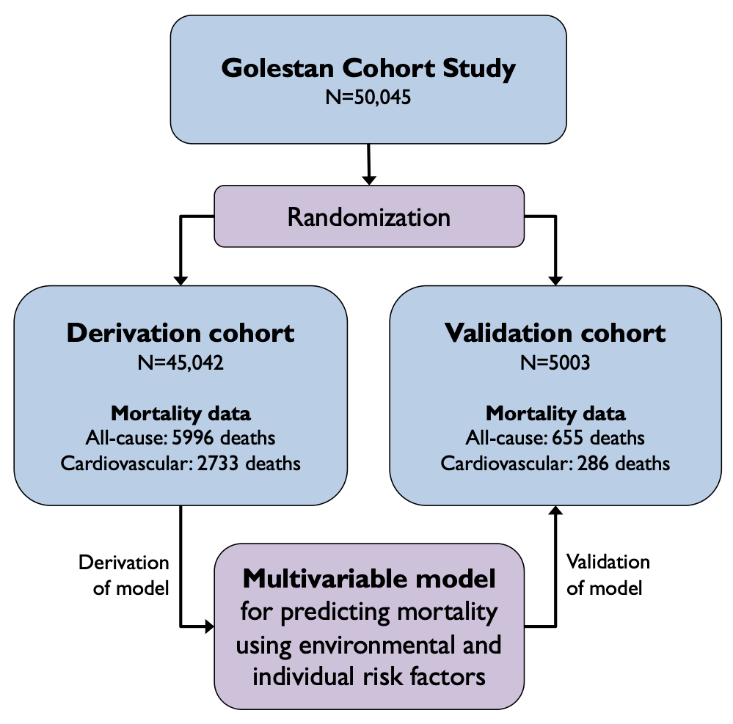


**S1 Fig: Summary of study design.** Enrollees in the Golestan Cohort Study were randomized into Derivation and Validation cohorts. The Derivation cohort was used to generate a multivariable survival model incorporating spatial environmental and individual factors to predict all-cause and cardiovascular mortality. This model was subsequently tested on the separate Validation cohort.


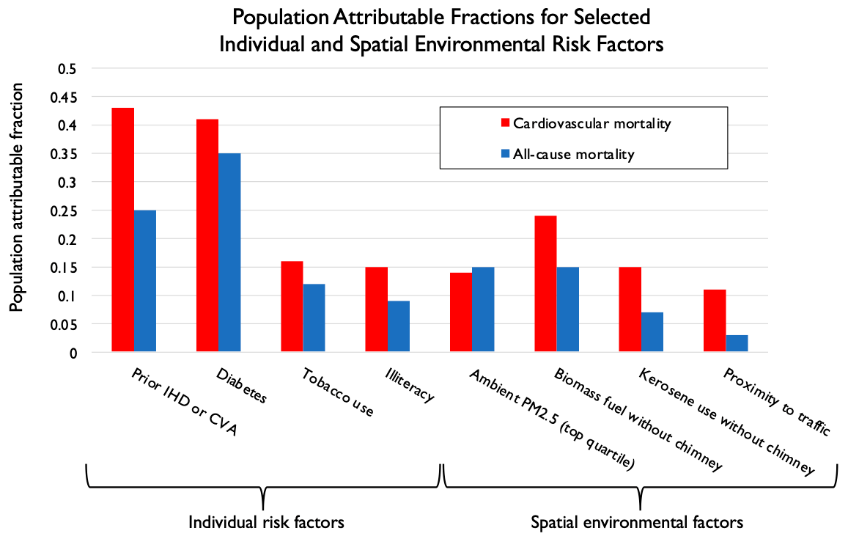


**S2 Fig: Population-attributable fractions (PAFs) for selected individual risk factors and spatial environmental factors**. PAFs estimate the fraction of total disease risk in the population that would be eliminated (or added) if the risk factor were eliminated from the population (i.e., if all individuals with that risk factor were moved to the reference category). IHD = ischemic heart disease. CVA = cerebrovascular accident (stroke). PM_2.5_ = fine particulate matter air pollution.

| **S1 Table: Data sources for spatial modeling of environmental risk factors** | |
| --- | --- |
| **Environmental risk factor** | **Methodology** |
| **Ambient PM_2.5_ exposure** | Average annual exposures were assigned to geocodes according to a model combining aerosol optical depth from NASA MODIS, MISR, SeaWIFS satellite instruments with GEOS-Chem chemical transport model, and subsequently calibrated to global ground-based measurements using geographically weighted regression [2, 3]. |
| **Household fuel use and ventilation** | Fuel use patterns were documented for households at time of enrollment in the GCS [9]. |
| **Proximity to traffic** | Geocodes were categorized as within proximity to traffic if they were within 500m of a major highway, or within 100m of a minor highway, as defined by GCS collaborators based in Golestan [9]. The radius of exposure was larger for major roads with higher traffic throughput [4, 10, 11]. |
| **Proximity to health center** | Proximity was defined as the straight-line distance from each geocode to the nearest percutaneous coronary intervention center [9]. |
| **Neighborhood socioeconomic status** | Neighborhood socioeconomic status was assigned to each geocode based on the median socioeconomic score of all individuals enrolled at that geocode. Individual socioeconomic score was derived by applying multiple correspondence analysis (MCA) to appliance ownership data recorded in the GCS [1]. |
| **Local population density** | Population density was assigned to each geocode according to the LandScan model [6], combining spatial data, satellite imagery, and multi-variable dasymetric modeling. |
| **Light at night** | Average annual nighttime light exposure was assigned to each geocode according to light intensity estimates from the Operational Linescan System of the Defense Meteorological Satellite Program [7]. |
| **Local land use** | Local land use was assigned to geocodes according to the USGS global land cover survey and NLCD 92 land cover definitions [8] based on satellite imagery of local ecology. |

Methodologies used to develop high-resolution spatial exposure models for each of the studied environmental risk factors. Individuals were assigned exposures to each environmental risk factor based on these models. Data sources and methodologies are provided as citations. Please see Data Sharing Statement for information on how to access GCS-specific data.

| **S2 Table: Univariate hazard ratios for spatial environmental factors** | | |
| --- | --- | --- |
| **Spatial environmental factor** | **Hazard ratio [95% CI]** | |
|  | **Cardiovascular mortality All-cause mortality** | |
| Ambient PM_2.5_ (μg/m^3^, reference = quartile 1)  Quartile 2  Quartile 3  Quartile 4 | 1.02 [0.90, 1.17]  1.06 [0.93, 1.21]  1.05 [0.95, 1.18] | 1.01 [0.91, 1.11]  1.05 [0.96, 1.14]  0.96 [0.88, 1.05] |
| Household fuel use (reference = gas)  Biomass without chimney  Biomass with chimney  Kerosene without chimney  Kerosene with chimney  Mixed fuel use | 1.52 [1.14, 2.02]  1.19 [0.99, 1.42]  1.13 [1.00, 1.28]  0.86 [0.75, 0.98]  1.06 [0.91, 1.24] | 1.56 [1.28, 1.90]  1.31 [1.16, 1.48]  1.18 [1.09, 1.28]  0.94 [0.86, 1.03]  1.04 [0.93, 1.16] |
| Proximity to traffic (reference = no) | 1.06 [0.98, 1.14] | 0.95 [0.95, 1.00] |
| Distance to PCI center (per 10 km) | 1.02 [1.01, 1.03] | 1.02 [1.01, 1.03] |
| Neighborhood SES score | 0.58 [0.43, 0.79] | 0.43 [0.35, 0.53] |
| Population density (per 1000 persons/km^2^) | 1.00 [0.99, 1.02] | 0.99 [0.98, 1.00] |
| Light at night intensity | 1.00 [1.00, 1.00] | 1.00 [1.00, 1.00] |
| Land use (reference = urban)  Cropland  Shrubland  Grassland/savanna/barren | 0.98 [0.89, 1.07]  1.13 [0.99, 1.30]  1.03 [0.89, 1.19] | 1.13 [1.06, 1.20]  1.16 [1.06, 1.28]  1.15 [1.04, 1.27] |

Univariate (unadjusted) hazard ratios for cardiovascular and all-cause mortality associated with eight SEFs. Three SEFs predicted both cardiovascular and all-cause mortality: household fuel use (biomass without chimney, kerosene without chimney), distance to PCI center, and neighborhood SES. Additionally, land use (cropland, shrubland, or grassland/savanna/barren) predicted of all-cause mortality in the univariate model. In the multivariable model incorporating all eight SEFs as well as individual factors, neighborhood SES and land use mix were no longer demonstrated clear relationships with mortality (**Table 4**). PM_2.5_ = fine particulate matter air pollution. PCI = percutaneous coronary intervention. SES = socioeconomic status.

| **S3 Table: Population attributable fractions for individual and spatial environmental factors** | | |
| --- | --- | --- |
| ***Individual risk factors*** | **Cardiovascular mortality** | **All cause mortaliity** |
| Male sex (reference = female) | 0.40 | 0.38 |
| Turkmen ethnicity (reference = all others) | 0.05 | 0.09 |
| Married (reference = all others) | -0.15 | -0.16 |
| Illiteracy (reference = all others) | 0.15 | 0.09 |
| SES (reference = quartile 1) |  |  |
| Quartile 2 | -0.08 | -0.15 |
| Quartile 3 | -0.30 | -0.24 |
| Quartile 4 | -0.40 | -0.39 |
| Physical activity (reference = tertile 3) |  |  |
| Tertile 1 | 0.26 | 0.28 |
| Tertile 2 | 0.10 | 0.21 |
| History of IHD or CVA (reference = none) | 0.43 | 0.25 |
| History of diabetes (reference = none) | 0.41 | 0.35 |
| History of hypertension (reference = none) | 0.42 | 0.25 |
| Current tobacco use (reference = never) | 0.16 | 0.12 |
| Former tobacco use (reference = never) | 0.20 | 0.19 |
| History of opium use (reference = never) | 0.28 | 0.26 |
| History of alcohol use (reference = never) | 0.01 | 0.06 |
| ***Spatial environmental factors*** |  |  |
| Ambient PM_2.5_ (μg/m^3^, reference = quartile 1) |  |  |
| Quartiile 2 | 0.09 | 0.06 |
| Quartile 3 | 0.08 | 0.07 |
| Quartile 4 | 0.14 | 0.15 |
| Houseehold fuel use (reference = gas) |  |  |
| Biomass without chimney | 0.24 | 0.15 |
| Biomass with chimney | 0.09 | 0.04 |
| Kerosene without chimney | 0.15 | 0.07 |
| Kerosene with chimney | -0.02 | -0.02 |
| Mixed fuels | 0.02 | -0.02 |
| Proximity to traffic (reference = no) | 0.11 | 0.03 |
| Land use (reference = urban) |  |  |
| Cropland | -0.07 | 0.06 |
| Shrubland | 0.03 | 0.04 |
| Grassland/savanna/barren | -0.09 | 0.02 |

Population attributable fractions (PAFs) for all categorical individual risk factors and spatial environmental factors. PAFs estimate the fraction of total disease risk in the population that would be eliminated (or added) if the risk factor were eliminated from the population (i.e., if all individuals with that risk factor were moved to the reference category). SES = socioeconomic status. IHD = ischemic heart disease. CVA = cerebrovascular accident (stroke). PM_2.5_ = fine particulate matter air pollution.

**References**

1. Islami F, Kamangar F, Nasrollahzadeh D, Aghcheli K, Sotoudeh M, Abedi-Ardekani B, et al. (2009). Socio-economic status and oesophageal cancer: results from a population-based case-control study in a high-risk area. Int J Epidemiol 38(4):978-88.

2. van Donkelaar A, Martin RV, Brauer M, Boys BL. Use of satellite observations for long-term exposure assessment of global concentrations of fine particulate matter. Environ Health Perspect. 2015;123(2):135-43. doi: 10.1289/ehp.1408646. PubMed PMID: 25343779; PubMed Central PMCID: PMCPMC4314252.

3. Atmospheric Composition Analysis Group (2019). Global Estimates of All Composition Surface PM2.5. Accessed December 10, 2019. Available at: <https://sites.wustl.edu/acag/datasets/surface-pm2-5/#V5.GL.02>.

4. Brauer M, Hoek G, van Vliet P, Meliefste K, Fischer P, Gehring U, et al. Estimating long-term average particulate air pollution concentrations: application of traffic indicators and geographic information systems. Epidemiology. 2003;14(2):228-39. doi: 10.1097/01.EDE.0000041910.49046.9B. PubMed PMID: 12606891.

5. Ahmadi A, Soori H, Mehrabi Y, Etemad K. Spatial analysis of myocardial infarction in Iran: National report from the Iranian myocardial infarction registry. J Res Med Sci. 2015;20(5):434-9. PubMed PMID: 26487871; PubMed Central PMCID: PMCPMC4590197.

6. Laboratory. ORN. LandScan. 2017. Available from: <http://web.ornl.gov/sci/landscan/>.

7. NOAA National Centers for Environmental Information (2016). Nighttime lights composites for the years 1992-2013 from DMSP satellite data. Accessed December 10, 2019. Available at: <http://ngdc.noaa.gov/eog/dmsp/downloadV4composites.html>

8. Friedl, M. & D Sulla-Menashe (2019). MCD12Q1 MODIS/Terra+Aqua Land Cover Type Yearly L3 Global 500m SIN Grid V006 [Data set]. NASA EOSDIS Land Processes DAAC. Accessed December 12, 2019. Available at: <https://lpdaac.usgs.gov/products/mcd12q1v006/>.

9. Pourshams A, Khademi H, Malekshah AF, Islami F, Nouraei M, Sadjadi AR, et al. Cohort Profile: The Golestan Cohort Study--a prospective study of oesophageal cancer in northern Iran. Int J Epidemiol. 2010;39(1):52-9. doi: 10.1093/ije/dyp161. PubMed PMID: 19332502; PubMed Central PMCID: PMCPMC3709199.

10. Hoek G, Beelen R, De Hoogh K, Vienneau D, Gulliver J, Fischer P, et al. A review of land-use regression models to assess spatial variation of outdoor air pollution. Atmospheric environment. 2008;42(33):7561-78.

11. Adar SD, Kaufman JD. Cardiovascular disease and air pollutants: evaluating and improving epidemiological data implicating traffic exposure. Inhal Toxicol. 2007;19 Suppl 1:135-49. doi: 10.1080/08958370701496012. PubMed PMID: 17886061.
